# Fibulin-2 expression associates with vascular invasion and patient survival in breast cancer

**DOI:** 10.1371/journal.pone.0249767

**Published:** 2021-04-09

**Authors:** Tor A. Klingen, Ying Chen, Hans Aas, Elisabeth Wik, Lars A. Akslen

**Affiliations:** 1 Centre for Cancer Biomarkers CCBIO, Department of Clinical Medicine, Section for Pathology, University of Bergen, Bergen, Norway; 2 Department of Pathology, Vestfold Hospital Trust, Tønsberg, Norway; 3 Department of Pathology, Oslo University Hospital, Oslo, Norway; 4 Department of Surgery, Vestfold Hospital Trust, Tønsberg, Norway; 5 Department of Pathology, Haukeland University Hospital, Bergen, Norway; Seoul National University College of Pharmacy, REPUBLIC OF KOREA

## Abstract

Stromal elastosis is related to good prognosis in breast cancer and fibulin-2 helps to stabilize elastic fibers in basement membranes. Here, we examined the level of perivascular fibulin-2 expression in relation to elastosis content, vascular invasion, molecular subtypes, tumour detection mode, and patient prognosis in breast cancer. We performed a population based retrospective study of invasive breast cancers from the Norwegian Breast Screening Program (Vestfold County, 2004–2009) including 200 screen-detected and 82 interval cancers. Perivascular fibulin-2 staining was semi-quantitatively graded based on immunohistochemistry (1–3) and dichotomized as high expression (grade 2–3) and low expression (grade 1). Elastosis content was graded on a 4-tiered scale and dichotomized as high (score 3) and low (score 0–2) expression, whereas lymphatic (LVI) and blood vessel invasion (BVI) were recorded as absent or present by immunohistochemistry. High perivascular fibulin-2 expression was strongly related to stromal elastosis (p<0.001), and inversely associated with BVI and LVI (p<0.001 for both). High fibulin-2 was associated with luminal breast cancer subgroups (p<0.001) and inversely with interval cancers compared with screen-detected tumours (p<0.001). By univariate analysis, low perivascular fibulin-2 was associated with reduced recurrence-free survival (p = 0.002) and disease specific survival (p = 0.019). Low perivascular fibulin-2 expression was strongly related to vascular invasion, low stromal elastosis, non-luminal breast cancer subtypes, interval presentation, and adverse prognosis.

## Introduction

Stromal elastosis, defined as dense aggregates of elastic fibers, is found in some neoplastic tissues and especially in malignant tumours of the breast [1], lung [2] and thyroid gland [3]. We previously reported that breast cancer stromal elastosis is associated with a favorable prognosis, low tumour cell proliferation by Ki67 expression, and mammography detection compared with interval cancer presentation in a population based setting [4]. In addition, high elastosis content correlated strongly with stellate tumour shape, low histological grade, and ER+/HER2– status [4]. However, whether and how elastosis is mechanistically involved in tumour development and prognosis in breast cancer is poorly understood.

Fibulins are calcium-binding glycoproteins that are linked to microfibrils in elastotic fibers, and fibulin-2 helps to stabilize elastic fibers in basement membranes [5, 6]. Immunohistochemical studies of fibulin-2 expression in normal human breast tissue have shown strong staining, especially surrounding vessels and ducts [7]. Based on this, we hypothesized that reduced perivascular fibulin-2 expression is associated with improved tumour cell accessibility into breast cancer vessels and other features of aggressive tumours. Notably, the relationship between levels of fibulin-2, vascular invasion and detection mode (screen-detected versus interval breast cancer) has not been previously investigated. Also, to our knowledge, there is no information on how fibulin-2 protein expression, in particular around vessel structures, relates to prognosis in breast cancer.

The aim of this study was to establish whether perivascular fibulin-2 expression is associated with patient prognosis in breast cancer, and to see whether this expression pattern is related to vascular invasion, stromal elastosis, molecular subtypes, and tumour detection mode.

## Materials and methods

### Patient series

Patients were included from Vestfold County in Eastern Norway. Vestfold comprises 5% of the Norwegian population with around 230,000 inhabitants. The Norwegian Breast Cancer Screening Program involves biannual mammography in the age-group 50–69 years, and was implemented in this county in 2004. A total of 37,977 women participated during the study period January 2004 –December 2008, with attendance rates of 71% and 76% during the first two screening rounds. During this period, 202 invasive screen-detected cancers and 83 invasive interval breast tumours were diagnosed after the prevalent and subsequent rounds.

Information on completeness was received from the Cancer Registry of Norway. The patients (age 50–69 years at diagnosis) were predominantly Caucasians living in Vestfold County. Ten patients (7 interval cases, 3 screening cases) were excluded because only core biopsies were available. Three other patients were also excluded due to the following reasons: 1 screen-detected cancer had no residual tumour tissue for further investigation, 1 screen-detected tumour was diagnosed as a malignant phyllodes tumour and 1 patient with interval cancer suffered from multiple metastases at the time of diagnosis with no biopsy or breast surgery having been performed. Four patients (two screening-detected cases and two interval cases) had cancers in both breasts, and the Nottingham Prognostic Index (NPI) was used to select the tumour with the worst prognosis in three of them. The fourth patient showed identical NPI for both tumours, so the tumour with the highest Ki67 score was selected. Thus, a total of 272 patients (197 screen-detected and 75 interval cases) were finally included. Clinical data (tumour stage and survival time) were recorded from patients’ journals with last status recorded in August 2017. The median follow-up time was 126 months (range 2–168 months). Primary treatment of the patients included 199 resections (73%) and 73 mastectomies (27%). In addition, 213 patients (82%) received radiation, 152 (59%) received endocrine treatment and 70 (27%) received chemotherapy. Treatment information for 12 patients (4.4%) was unavailable.

### Ethics statement

The study was approved by the Regional Ethics Committee of Eastern Norway (reference #2018/1102). The Regional Ethics Committee waived the requirement for informed consent. All histopathological and clinical data used were deidentified in this study.

### Histopathological data

Tumours were classified as invasive carcinoma of no special type (NST) or special type carcinomas (lobular, other types). Histological grading was done according to the Nottingham criteria [8]. Tumour diameter was measured microscopically in mm, and lymph node status was included from the pathology report.

### Immunohistochemistry

Whole tumour sections were used for fibulin-2 staining. Slides were placed on the Ventana Benchmark Ultra automated immuno-stainer (Roche) and dewaxed. Heat-induced epitope retrieval was performed with Ventana`s Ultra CC1 retrieval solution for 56 minutes at 99°C followed by incubation with a rabbit polyclonal anti-fibulin-2 antibody (Thermo Fisher, product number PA5-51665 and lot number A35146) at a 1:200 dilution in Ventana Antibody Diluent (251–018) for 32 minutes at 36°C. Presence of the antigen was visualized by using the OptiView DAB detection kit (Roche).

#### Evaluation of staining

**Fig 1:**
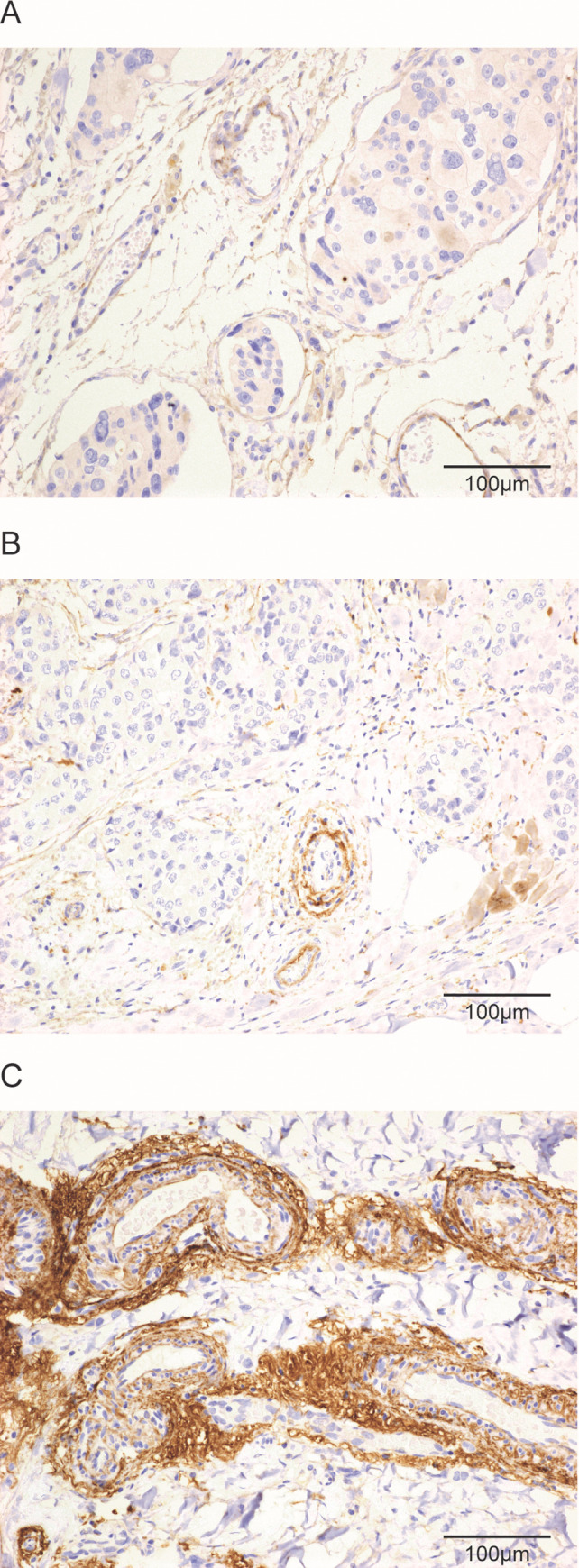
Histological images of perivascular fibulin-2 expression by immuno-histochemistry. Histological images of tumour tissue with absent or faint/barely perceptible peripheral staining in tumour vessels (A), weak to moderate staining (B), or strong/thick staining (C) for fibulin-2 in vessels at the tumour periphery (x200). Scale bars are shown on each image.

Fibulin-2 staining was detected around vessels (perivascular stroma), especially related to small vessels localized in the tumour periphery, close to the invasive border. Perivascular fibulin-2 staining was evaluated on the basis of the predominant staining pattern, and staining intensities in the tumour were graded in a semiquantitative manner from 1 to 3. Tumours were assigned staining grade 1 if fibulin-2 staining was absent or faint/barely perceptible in >50% of tumour vessels; grade 2, if weak to moderate peripheral staining was seen in >50% of tumour vessels; and grade 3, if stronger and thicker circumferential perivascular staining was observed in > 50% of tumour vessels (**Fig 1**).

For statistical analysis, perivascular fibulin-2 level was dichotomized as low-grade expression (staining grade 1) and high-grade expression (staining grade 2–3). To estimate inter-observer agreement for this grading system, 50 cases were examined by two pathologists (T.A.K., Y.C.) showing good agreement with kappa value of 0.70. Disagreement was only related to cases graded 2 or 3.

Some data from previous studies were included in this cohort for association analysis and comparisons. Briefly, the amount of elastosis was evaluated and graded by microscopy of standard sections in a semiquantitative manner from 0–3 according to Shivas and Douglas [9]. For statistical analysis, cases were divided into two categories: low elastosis (grade 0–2) with no or limited elastosis, and high elastosis content (grade 3) with extensive elastosis. Immunohistochemistry and evaluation of lymph vessel invasion (LVI) and blood vessel invasion (BVI) by D2-40 staining and CD31 staining, respectively, have previously been reported by us [10, 11], using whole sections. We recorded LVI to be present if tumour tissue was located within more than one D2-40 positive structure with weak or negative CD31 staining. On the other hand, BVI was reported when tumour emboli were detected in one or more CD31 positive and D2-40 negative vessels [10, 11]. Surrogate markers for molecular subtypes of breast cancer were defined and applied according to the St Gallen 2013 consensus [12]. The cut-off point for estrogen and progesterone receptors was 1% in the present study. Positivity for CK5/6 and/or P-cadherin was used to define basal-like differentiation [13, 14]. For Ki67 evaluation, 500 tumour cell nuclei were counted (hotspots) and the Ki67 positive fraction (%) was calculated.

### Statistical methods

Statistical analyses were performed using IBM SPSS Statistics, version 25.0 (Armonk, NY: IBM Corp.). Two-sided p-values of <0.05 were considered statistically significant. Associations between categorical variables were assessed by Pearson’s chi-square (χ^2^) test. In univariate Kaplan-Meier survival analyses (log-rank test for differences), disease recurrence and death from breast cancer were end-points (recurrence free and disease specific survival, RFS and DSS). Entry date was the time of diagnosis. Patients who died from other causes were censored at the date of death. The Cox´ proportional hazards method was used for multivariate survival analyses. Death from breast cancer was used as end-point. Variables were visually examined by log-minus-log plots to check proportionality assumptions. A multivariate analysis was conducted for perivascular fibulin-2 content together with standard prognostic variables. For statistical analysis, perivascular fibulin-2 amount was dichotomized as low-grade expression (staining grade 1) and high-grade expression (staining grade 2–3). After exclusion of a few cases (see above), a total of 272 patients were available for survival analyses in the current study.

## Results

### Staining patterns and distribution of fibulin-2

Fibulin-2 was predominantly expressed in the perivascular region. However, weak to strong fibulin-2 staining was also observed in the periphery of ductal carcinoma in situ (periductal staining), and in a few normal vessels and benign ducts in the tumour surroundings.

### High perivascular fibulin-2 expression associates with clinico-pathological characteristics of good prognosis

In our series, 201 cases (74%) showed high-grade perivascular fibulin-2 expression, whereas 71 cases (26%) showed low-grade staining. **Table 1** presents the basic clinico-pathological features in relation to amount of fibulin-2 around vessels in the study population. High perivascular fibulin-2 was related to predictors of good prognosis including low histological grade, ER and PR positivity, negative HER2 status and high Ki67 index (upper quartile; all p<0.001). Trends were also present for associations between perivascular fibulin-2 expression and small tumour size (< 2 cm) (p = 0.056), as well as negative nodal status (p = 0.068).

**Table 1:** Associations between perivascular fibulin-2 expression and clinico-pathological features (n = 272).

Variable		Perivascular fibulin-2			
		High	Low		
Histologic type	N	N (%)	N (%)	OR	P-value
Others	52	47 (90)	5 (10)	1	0.003
Carcinoma NST	220	154 (70)	66 (30)	4.0 (1.5–10.6)	
Tumour diameter					
< 2cm	210	161 (77)	49 (13)	1	0.056
≥ 2cm	62	40 (65)	22 (35)	1.8 (0.98–3.3)	
Histologic grade					
1	75	72 (96)	3 (4)	1	<0.001
2–3	197	129 (65)	68 (35)	12.3 (3.8–41.7)	
Lymph node status					
Negative	181	140 (77)	41 (23)	1	0.068
Positive	91	61 (67)	30 (33)	1.7 (0.9–3.0)	
ER					
Positive	239	191 (80)	48 (20)	1	<0.001
Negative	33	10 (30)	23 (70)	9.2 (4.1–20.5)	
PR					
Positive	181	147 (81)	34 (19)	1	<0.001
Negative	91	54 (59)	37 (41)	2.9 (1,7–5.2)	
HER 2 status					
Negative	249	195 (78)	54 (22)	1	<0.001
Positive	23	6 (26)	17 (74)	10.2 (3.8–27.2)	
Ki67					
Low	209	172 (82)	37 (18)	1	<0.001
High	63	29 (46)	34 (54)	5.5 (3.0–10.0)	

P-values were obtained using Pearson´s Chi square test. Perivascular fibulin-2 was graded on immunohistochemical slides.

### High perivascular fibulin-2 expression is related to tumour stromal elastosis and associates inversely with vessel invasion in breast cancer

Among the 272 cases in this study, 45 (17%) had high elastosis content, whereas 227 (83%) had low elastosis. We found a strong and significant relation between high stromal elastosis and high perivascular fibulin-2 expression (p<0.001; **Table 2**). Evaluation of vessel invasion revealed tumours that had either LVI (n = 45, 16%) or BVI (n = 18, 6%) or both LVI and BVI (n = 25, 9%), giving overall frequencies of 25% and 15% for LVI and BVI, respectively. High perivascular fibulin-2 was inversely associated with BVI and LVI (both p<0.001) as compared to tumours without BVI or LVI (**Table 2**).

**Table 2:** Association between perivascular fibulin-2 expression and elastosis, vessel invasion, basal-like tumours, triple negative tumours as well as detection mode (n = 272).

Variable			Perivascular fibulin-2			
			Low	High		
		N	N (%)	N (%)	OR	P-value
Elastosis	High	45	2 (3)	43 (96)	1	<0.001
	Low	227	69 (30)	158 (70)	9.4 (2.2–39.9)	
BVI	Negative	233	48 (21)	185 (79)	1	<0.001
	Positive	39	23 (59)	16 (41)	5.5 (2.7–11.3)	
LVI	Negative	207	40 (19)	167 (81)	1	<0.001
	Positive	65	31 (48)	34 (52)	3.8 (2.1–6.9)	
Basal-like	No	224	46 (21)	178 (79)	1	<0.001
	Yes	48	25 (52)	23 (48)	4.2 (2.2–8.1)	
Triple negative	No	251	57 (23)	194 (77)	1	<0.001
	Yes	21	14 (67)	7 (33)	6.8 (2.6–17.7)	
Detection mode	Screening	197	38 (19)	159 (81)	1	<0.001
	Interval	75	33 (44)	42 (56)	3.3 (1.8–5.9)	

P-values were obtained using Pearson´s Chi square test. Perivascular fibulin-2 expression was graded on immunohistochemical slides.

Abbreviations: BVI, blood vessel invasion; LVI, lymph vessel invasion; N, number of cases; OR, odds ratio.

### High perivascular fibulin-2 expression is associated with luminal breast cancer subgroups and inversely associated with basal-like phenotype and interval breast cancer

The cohort used in our study showed 53% Luminal A, 31% Luminal B (HER2-negative), 5% Luminal B (HER2-positive), 3% HER2-type, and 8%Triple-negative tumours. Perivascular fibulin-2 staining was significantly associated with molecular subtypes (P<0.001) (**Table 3**) according to the St Gallen 2013 consensus.

**Table 3:** Association between perivascular fibulin-2 expression and tumour subtypes (n = 272).

	Luminal A	Luminal B HER2-	Luminal B HER2+	HER2 type	Triple negative		Total
	N (%)	N (%)	N (%)	N (%)	N (%)	P-value	N
Total	144 (53)	84 (31)	14 (5)	9 (3)	21 (8)		272
**Perivascular fibulin-2**							
Low	18 (13)	22 (26)	10 (71)	7 (78)	14 (67)	<0.001	71
High	126 (87)	62 (74)	4 (29)	2 (22)	7 (33)		201

Number of cases (N) and % within molecular subgroups is given according to the St Gallen 2013 consensus. P-values were obtained using Pearson`s Chi square test. Perivascular fibulin-2 expression was graded on immunohistochemical slides.

HER2-positive and Triple-negative tumours demonstrated typically low perivascular fibulin-2 compared to Luminal A and Luminal B (HER2-negative tumours). Furthermore, cases with high perivascular fibulin-2 were inversely associated with basal-like tumours and interval cancers (both p< 0.001; **Table 2)**.

### Survival analyses

The median follow-up period for survivors was 126 months (range 2–168 months). Among 272 patients, distant metastases or local tumour recurrence was observed at follow-up in 42 patients, 32 patients died of breast cancer.

**Fig 2:**
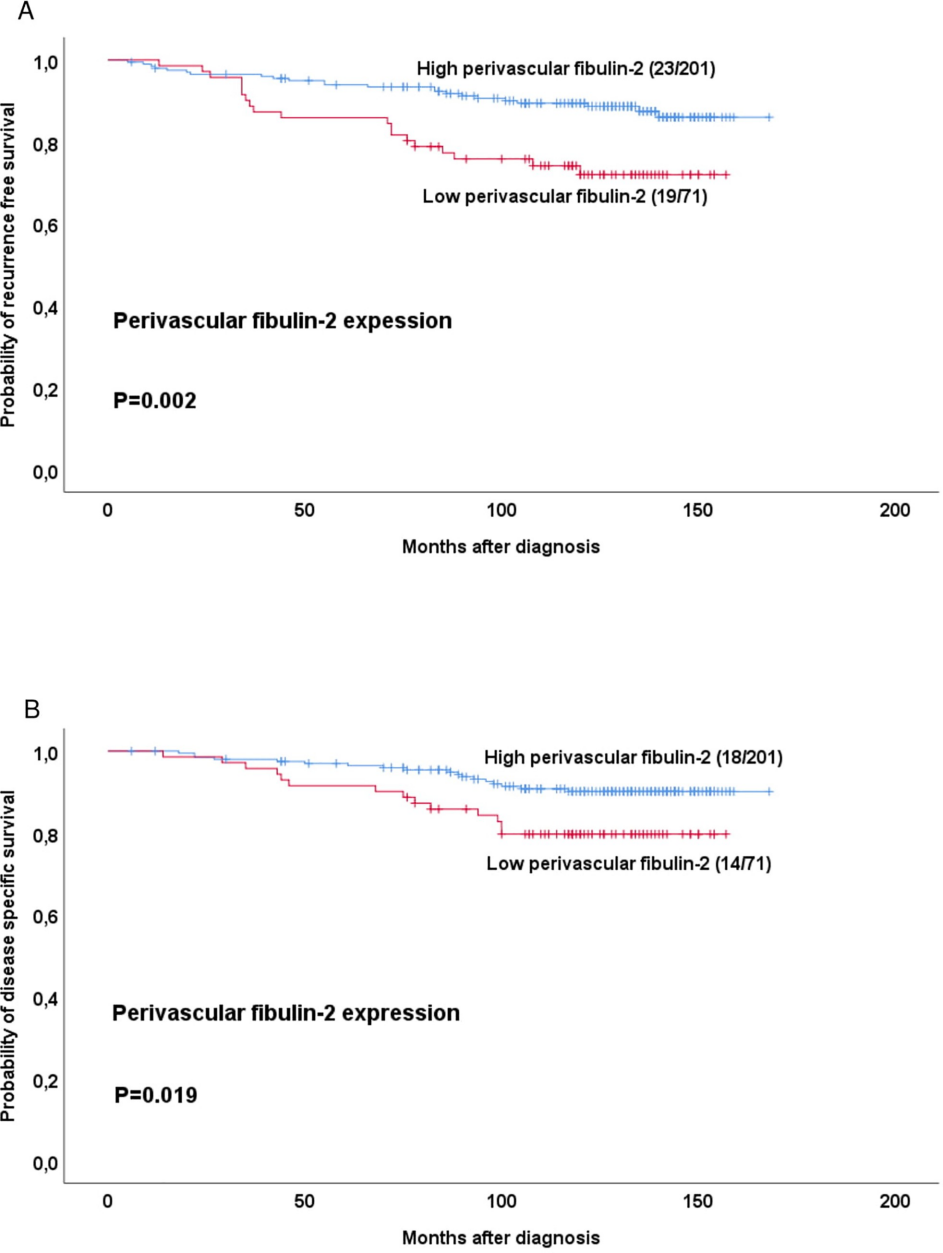
Estimated recurrence free and disease specific survival according to high-grade or low-grade perivascular fibulin-2 expression. Survival curves were estimated by the Kaplan-Meier method (with log-rank test for differences). For each category, number of events / total number of cases are given.

Univariate analysis revealed that low perivascular fibulin-2 (score 1) was associated with reduced recurrence-free survival (RFS) and disease specific survival (DSS), compared to high perivascular fibulin-2 (score 2–3; **Fig 2**).

Notably, perivascular fibulin-2 did not associate significantly with survival within the five St. Gallen subtypes when analyzed individually. To note, each subgroup had few cases and events. However, when Luminal A and Luminal B (HER2-negative) tumours were combined in one group and analyzed, low perivascular fibulin-2 was associated with reduced RFS (p = 0.001), but not DSS (p = 0.14).

Univariate analysis of vessel invasion with respect to RFS and DFS revealed that BVI and LVI were significantly associated with reduced RFS and DSS (all p≤0.005; **S1 Fig**).

We then divided the whole patient series into three groups based on BVI or LVI status (positive/negative) in combination with fibulin-2 expression levels (high/low), and performed univariate analysis on groups with: (1) BVI + or LVI+ and low perivascular fibulin-2, (2) BVI- or LVI- and high perivascular fibulin-2 and (3) all other cases (BVI and/or LVI positive with high perivascular fibulin-2 or BVI and/or LVI negative with low perivascular fibulin-2).

**Fig 3:**
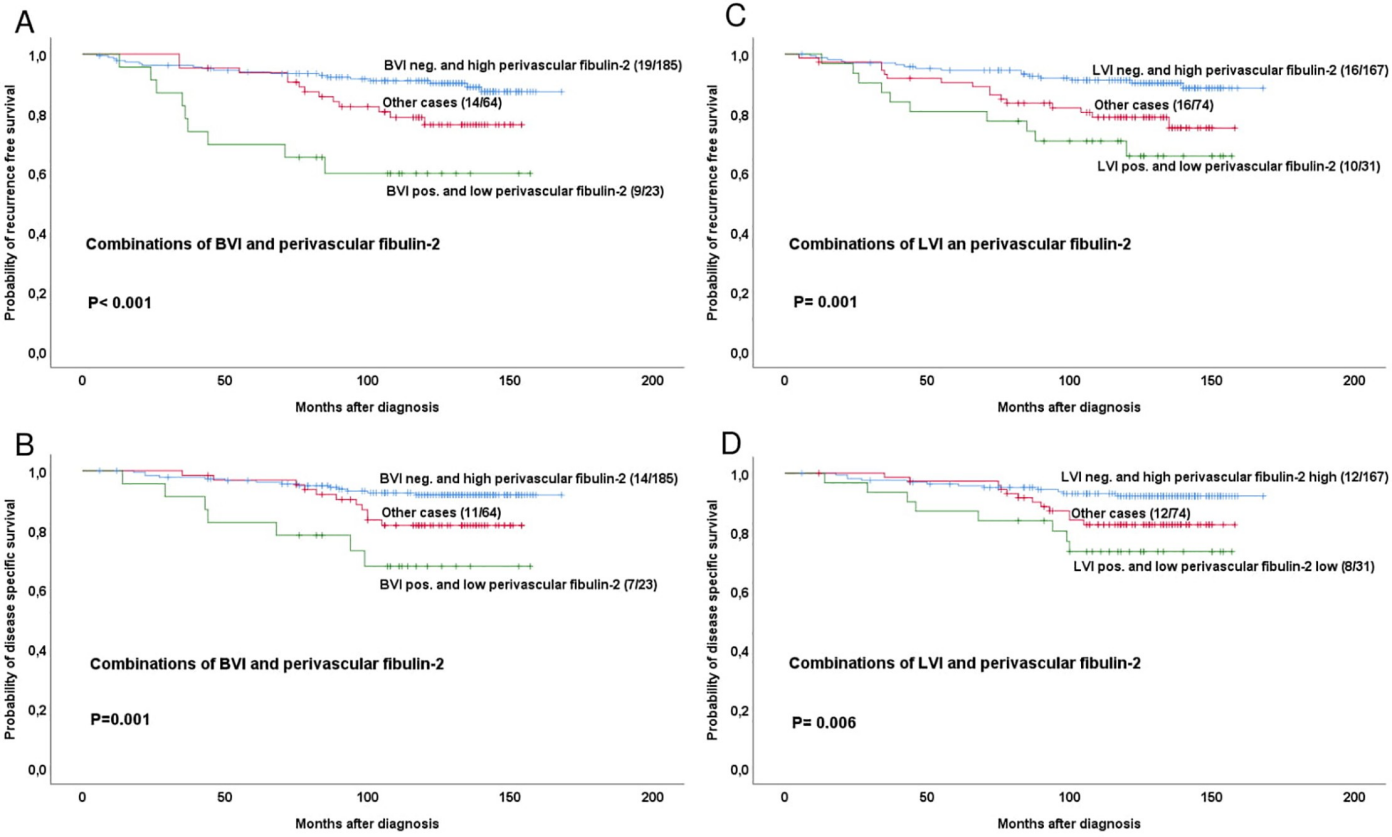
Estimated recurrence free and disease specific survival according to high-grade or low-grade perivascular fibulin-2 expression in combinations with BVI or LVI positive or negative cases. Survival curves are estimated by the Kaplan-Meier method (with log-rank test for differences). For each category, number of events / total number of cases are given. The category ‘other cases’ is defined as BVI positive with high perivascular fibulin-2 or BVI negative with low perivascular fibulin-2.

Significant differences for RFS and DSS were found for both BVI (**Fig 3A and 3B**) and LVI (**Fig 3C and 3D**)-based groups.

Multivariate analysis of RFS was performed for all cases by including perivascular fibulin-2 expression along with basic histopathologic markers such as tumour diameter, histologic grade and lymph node status. Here, fibulin-2 expression showed a borderline significant association with RFS (p = 0.052; **Table 4**).

**Table 4:** Univariate and multivariate recurrence free survival analysis (Cox`proportional hazard method) of pathological variables and perivascular fibulin-2 by immunohistochemistry (n = 272).

Variables	Categories	Univariate analysis		Multivariate analysis	
		HR (95%CI)	p-value	HR (95% CI)	p-value
Perivascular fibulin-2	High	1		1	
	Low	2.5 (1.4–4.6)	0.003	1.9 (0.99–3.5)	0.052
Tumour diameter	< 2cm	1		1	
	≥2cm	2.5 (1.3–4.5)	0.003	1.3 (0.7–2.5)	0.431
Histologic grade	1	1		1	
	2–3	4.0 (1.4–11.2)	0.008	2.5 (0.9–7.6)	0.092
Lymph node status	Negative	1		1	
	Positive	4.0 (2.1–7.3)	<0.001	3.2 (1.7–7.6)	<0.001

Abbreviations: HR, hazard ratio; 95% CI; 95% confidence interval.

When including age (as a continuous variable) in addition to fibulin-2 expression along with tumour diameter, histologic grade and lymph node status in an alternative multivariate model, fibulin-2 was significant (HR = 1.88; 95% CI 1.00–3.54; p = 0.050) in addition to lymph node status (HR = 3.27; 95% CI 1.71–6.27; p<0.0005), whereas tumour diameter (p = 0.56), histologic grade (p = 0.09) and age (p = 0.10) were not significant in this series.

For comparison and validation of the cohort, a multivariate analysis of standard prognostic variables alone showed independent impact for histologic grade (p = 0.026) and lymph node status (p< 0.001), but not for tumour diameter in this population- based cohort (S1 Table).

Multivariate analysis was also done for the same basic histopathologic factors in addition to perivascular fibulin-2 expression within the group of luminal (HER-2 negative) tumours. Low perivascular fibulin-2 in this subgroup showed a weaker association with shorter RFS (p = 0.12; (S2 Table) compared with low perivascular fibulin-2 in the whole series.

## Discussion

Previous studies have shown that fibulin-2 exhibits a high affinity for elastin [15]. The present study indicates a strong association between fibulin-2 expression and high amount of elastotic stroma in breast tumours. Since elastosis is a positive prognostic factor in breast cancer, our data are in line with findings that fibulin-2 expression reduces cell motility as well as invasion capacities in breast cancer cell lines [16]. Hypothetically, low elastosis content, and therefore a low fibulin-2 content, may facilitate tumour cell migration and invasion. Furthermore, fibulin-2 binds several proteins including fibronectin, laminin and nidogen, which act as scaffolds in basement membranes [17]. To speculate, a reduced anchoring of fibulin-2 could destabilize basement membranes and enhance the ability of tumour cells to migrate and invade through the ECM.

Although other fibulins might also interact with elastic fibers [18–20], we decided to use fibulin-2 in this study because of the perivascular staining pattern observed in normal breast tissue [7]. As a novel observation, we found a strong association between low (reduced) perivascular fibulin-2 content and both blood vascular invasion (BVI) and lymphatic involvement (LVI). Low fibulin-2 expression may possibly enhance metalloproteinase (MMP) activity, and thereby contribute to vessel invasion as seen in our study. In line with this, Fontanil et al. demonstrated that fibulin-2 might exert its tumour suppressor function by interaction with ADAMTS-12, a secreted MMP, while ADAMTS-12 may elicit pro-tumour effects in the absence of fibulin-2 [21]. MMPs induce proteolytic degradation of basement membranes and promote breast carcinoma intravasation in leaky vessels [22].

In contrast to a suppressive role in breast cancer, experimental studies from other organs have suggested that fibulin-2 may contribute to metastases and invasion in lung and pancreatic adenocarcinoma cell lines [23, 24], suggesting organ specific properties of fibulin-2. Because of this context dependence, fibulin-2 may also be of importance to metastases in breast cancer and might potentially have a different role. Fibulin-2 promotes cross-linking of collagen and migration of tumour cells in primary lung adenocarcinoma [23], and it would be of interest to explore its role in the metastatic setting. Interestingly, reduced levels of fibulin-5 in breast cancer have been reported to be required for metastasis formation in the liver and lung by suppressing the activity of MMP9, a protease that remodels the ECM during metastatic niche formation and promotes metastatic outgrowth [20].

It has been shown that differences in prognostic factors such as tumour diameter, histologic grade and lymph node status do not fully predict the difference between screen-detected and interval breast cancers [25, 26]. This is consistent with the observations that detection mode is an independent prognostic factor [27, 28]. We have previously reported a strong association between BVI and interval breast cancer [11]. The present study indicates a link between low perivascular fibulin-2 expression and interval breast cancer. Interval-detected tumours are more often basal-like and HER2-positive [27, 29], and demonstrate a stiffer collagen stroma compared to screen-detected tumours [30]. Importantly, expression of matrix remodeling genes such as MMPs and collagen cross-linkers is predictive of a poor prognosis for breast cancer patients [31]. To speculate, interval breast cancers with low perivascular fibulin-2 content may have more collagen cross-linking and collagen linearization. This may help tumour cells to migrate into vascular channels, as an early step of metastatic spread.

In conclusion, this population-based study indicates a strong relation between reduced perivascular fibulin-2 content and vascular invasion as well as tumour detection during screening intervals. Furthermore, low elastosis content and low fibulin-2 expression are strongly related. The presence of low perivascular fibulin-2 was more often observed in non-luminal subgroups and basal-like breast cancer and was associated with adverse prognosis.

## Supporting information

S1 Fig Estimated recurrence free and disease specific survival according to presence of blood vessel invasion (BVI) and lymphatic vessel invasion (LVI). Survival curves are estimated by the Kaplan-Meier method (with log-rank test for differences). For each category, number of events / total number of cases are give.
(TIFF)

S1 Table Univariate and multivariate recurrence free survival analysis (Cox`proportional hazards method) of traditional pathological variables (n = 272). *Abbreviations*: HR, hazard ratio; 95% CI; 95% confidence interval.
(DOCX)

S2 Table Univariate and multivariate recurrence free survival analysis (Cox`proportional hazards method) of pathological variables and perivascular fibulin-2 expression by immunohistochemistry in luminal (HER2 negative) tumours (n = 228). *Abbreviations*: HR, hazard ratio; 95% CI; 95% confidence interval.
(DOCX)
