# Supplementary figures and images for: Fibulin-2 expression associates with vascular invasion and patient survival in breast cancer
Source: PLoS One. 2021 Apr 9;16(4):e0249767. doi: 10.1371/journal.pone.0249767 (PMC8034712; doi:10.1371/journal.pone.0249767)

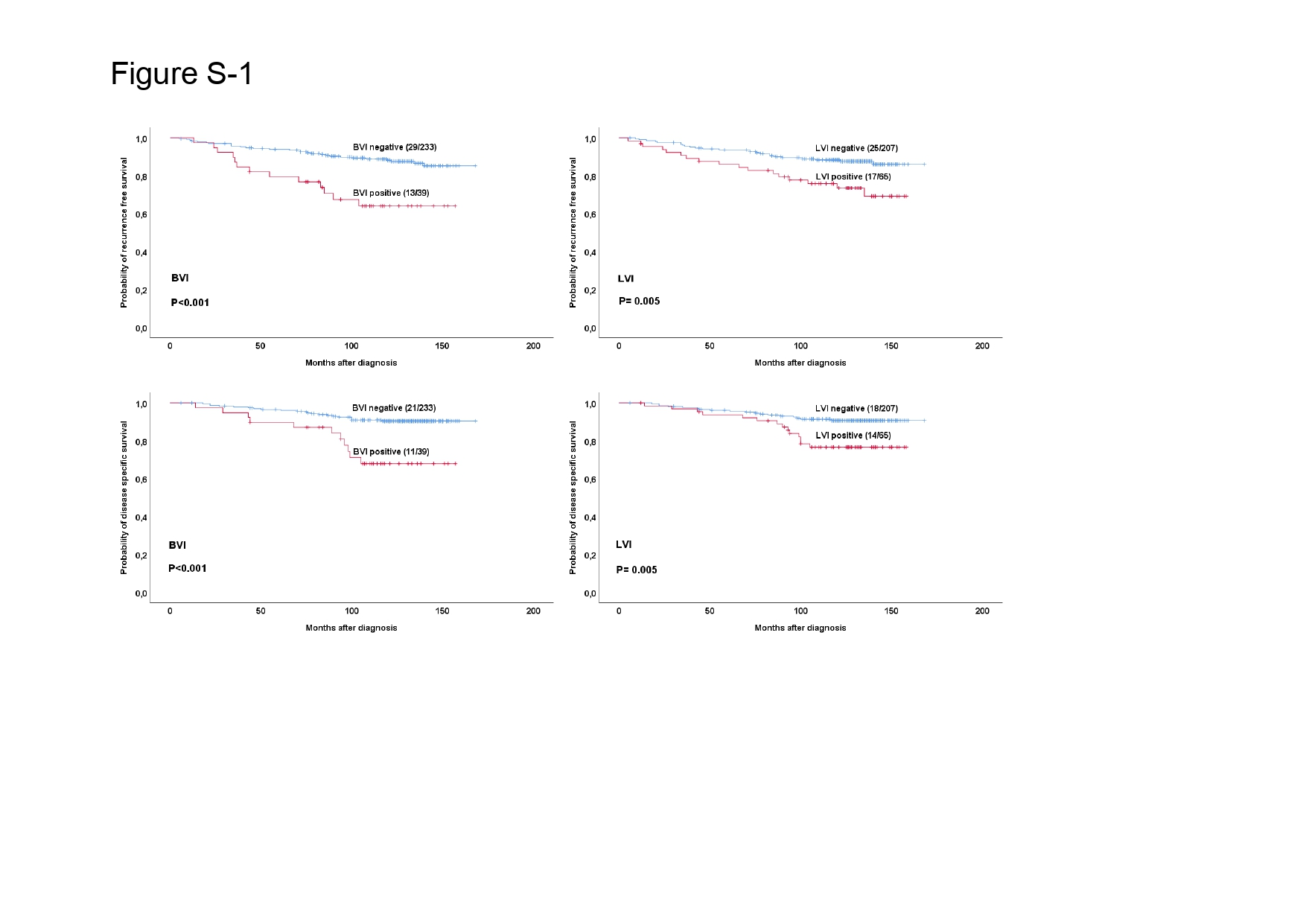

Supplement: S1 Fig — Survival curves are estimated by the Kaplan-Meier method (with log-rank test for differences). For each category, number of events / total number of cases are give. (TIFF) [file pone.0249767.s001.tiff]
