# Supplementary material for: Fibulin-2 expression associates with vascular invasion and patient survival in breast cancer
Source: PLoS One. 2021 Apr 9;16(4):e0249767. doi: 10.1371/journal.pone.0249767 (PMC8034712; doi:10.1371/journal.pone.0249767)
Supplement: S1 Table — Abbreviations: HR, hazard ratio; 95% CI; 95% confidence interval. (DOCX) [file pone.0249767.s002.docx]

| **S1 Table.** Univariate and multivariate recurrence free survival analysis (Cox`proportional hazard method) of traditional pathological variables (n=272) | | | | | |
| --- | --- | --- | --- | --- | --- |
| Variables | Categories | Univariate analysis | | Multivariate analysis | |
|  |  | HR (95%CI) | p-value | HR (95% CI) | p-value |
|  |  |  |  |  |  |
| Tumour diameter | < 2cm | 1 |  | 1 |  |
|  | ≥2cm | 2.5 (1.3-4.5) | 0.003 | 1.3 (0.7-2.4) | 0.483 |
| Histologic grade | 1 | 1 |  | 1 |  |
|  | 2-3 | 4.0 (1.4-11.2) | 0.008 | 3.3 (1.1-9.5) | 0.026 |
| Lymph node status | Negative | 1 |  | 1 |  |
|  | Positive | 4.0 (2.1-7.3) | <0.001 | 3.5 (1.8-6.7) | <0.001 |
| Abbreviations: HR, hazard ratio; 95% CI; 95% confidence interval. | | | | | |
