# Supplementary material for: Fibulin-2 expression associates with vascular invasion and patient survival in breast cancer
Source: PLoS One. 2021 Apr 9;16(4):e0249767. doi: 10.1371/journal.pone.0249767 (PMC8034712; doi:10.1371/journal.pone.0249767)
Supplement: S2 Table — Abbreviations: HR, hazard ratio; 95% CI; 95% confidence interval. (DOCX) [file pone.0249767.s003.docx]

| **S2 Table**. Univariate and multivariate recurrence free survival analysis (Cox`proportional hazards method) of pathological variables and perivascular fibulin-2 expression by immunohistochemistry in luminal (HER2 negative) tumours (n=228). | | | | | |
| --- | --- | --- | --- | --- | --- |
| **Variables** | **Categories** | **Univariate analysis** | | **Multivariate analysis** | |
|  |  | HR (95%CI) | p-value | HR (95% CI) | p-value |
|  |  |  |  |  |  |
| Perivascular fibulin-2 | High | 1 |  | 1 |  |
|  | Low | 2.6 (1.3- 5.5) | 0.009 | 1.8 (0.85-3.9) | 0.12 |
|  |  |  |  |  |  |
| Tumour diameter | <2cm | 1 |  | 1 |  |
|  | ≥2cm | 2.8 (1.4-5.7) | 0.003 | 1.6 (0.7-3.4) | 0.25 |
|  |  |  |  |  |  |
| Histologic grade | 1 | 1 |  | 1 |  |
|  | 2-3 | 3.8 (1.3-10.8) | 0.013 | 2.4 (0.8-7.3) | 0.12 |
|  |  |  |  |  |  |
| Lymph node status | Negative | 1 |  | 1 |  |
|  | Positive | 3.8 (1.8-7.6) | <0.001 | 2.8 (1.3-5.9) | 0.009 |
| Abbreviations: HR, hazard ratio; 95% CI; 95% confidence interval; | | | | | |
